# Testosterone reactivity moderates the association between reactive aggression and suicidality in major depression

**DOI:** 10.1192/j.eurpsy.2026.10212

**Published:** 2026-07-31

**Authors:** H. E. Baki, O. Aktan, E. Bodur, K. Başar

**Affiliations:** 1 Department of Psychiatry, Fatsa State Hospital, Ordu; 2 Department of Medical Biochemistry; 3 Department of Psychiatry, Hacettepe University Faculty of Medicine, Ankara, Türkiye

## Abstract

**Introduction:**

Reactive aggression (RA) is associated with suicidality; however, this relation has not been tested with behavioral measures of RA. Earlier, RA has been examined in relation to testosterone (T) and cortisol (C). In individuals with suicidal behavior, dysregulated cortisol responses have been reported, whereas the role of testosterone reactivity remains unclear.

**Objectives:**

To investigate the associations of suicidal ideation (SI) and suicide attempt (SA) with laboratory-measured RA, baseline T and C levels and their reactivity, and clinical variables, and to test whether testosterone and cortisol reactivity moderate the association between RA and suicidality (SI/SA) in patients with major depression.

**Methods:**

Patients with major depression (n=95; HAM-D≥14) were classified based on the Columbia-Suicide Severity Rating Scale into three groups: major depression (MD; n=42), suicidal ideation (SI; ideation in the past month; n=31), and suicide attempt (SA; attempt in the past month; n=22). Participants completed a 10-min Point Subtraction Aggression Paradigm (PSAP) for behavioral assessment of RA. Saliva samples were collected pre-task and 15 min post-task; T and C were measured by ELISA. Reactivity (ΔT%, ΔC%) was the percent change from baseline. STAXI-State Anger was administered pre- and post-task. Additional self-report measures included BPAQ, BIS-11, Fava AAQ, and INQ (perceived burdensomeness and thwarted belongingness). Analyses used a general linear model (GLM) and multinomial logistic regression; moderation was evaluated via interaction terms.

**Results:**

Reactive aggression differed by group (*p*=.004, *η*²=.12), higher in SA than in SI and MD. State anger increased during PSAP (*p*<.001). Baseline hormone levels and hormonal reactivity (ΔT%, ΔC%) did not differ across groups (all p>.10). In the GLM predicting RA (sex, group, baseline T/C, ΔT%, ΔC%, group × ΔT%), the group × ΔT% interaction was significant (*F*=3.63, *p*=.031, *η*²=.08), driven by the SA group. Within SA, ΔT% positively predicted RA (*B*=0.034, *p*=.008) and explained 14.4% of the variance (Image 1). In the multinomial model, higher reactive aggression, higher depression severity, greater perceived burdensomeness, and lower baseline T were significant predictors of SA. SI was only associated with perceived burdensomeness. Adding RA × ΔT% to the model improved model fit (Nagelkerke *R²*=.583), and the interaction significantly predicted SA vs MD.

**Image 1:**
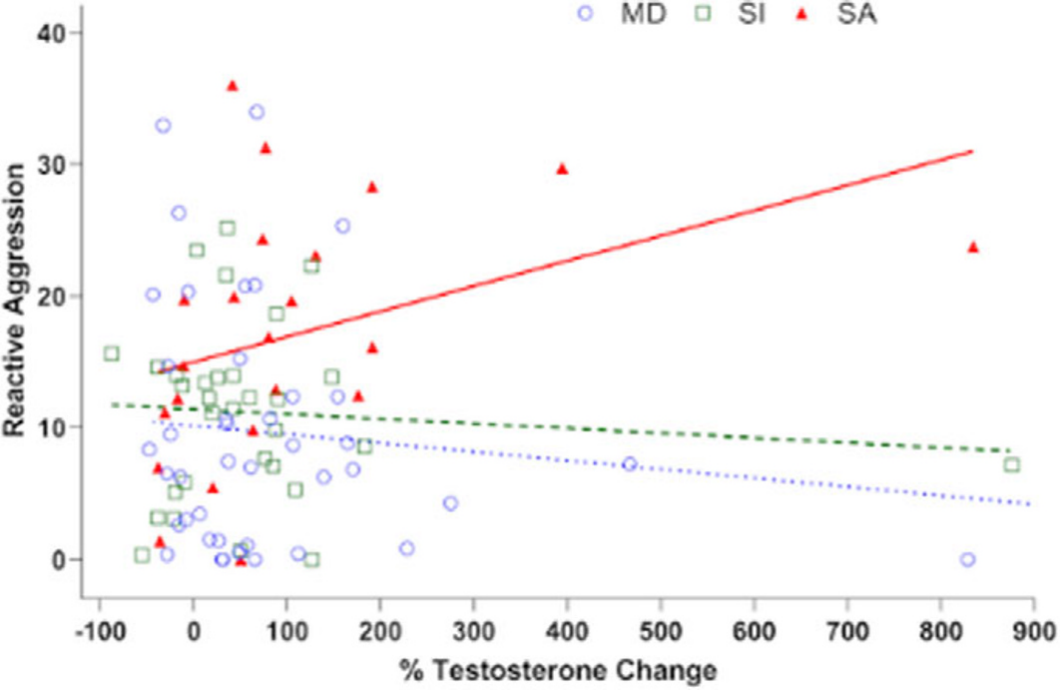
Image 1: Long description.

**Conclusions:**

Major depression patient with a recent suicide attempt history showed higher reactive aggression on a behavioral task compared to those without an attempt history. In patients with a suicide attempt, greater task-related testosterone increases were associated with higher RA. Moreover, the finding that the interaction between RA and testosterone reactivity predicted suicide attempts may have implications for understanding hormone–behavior patterns associated with suicide risk.

**Disclosure of Interest:**

H. Baki Grant / Research support from: Hacettepe University Scientific Research Projects Coordination Unit. Project Number: 19612., O. Aktan: None Declared, E. Bodur: None Declared, K. Başar: None Declared

## Long descriptions

### Image 1: Long description

The x-axis represents percent testosterone change ranging from negative 100 to 900. The y-axis represents reactive aggression ranging from 0 to 40. The data is categorized into three groups.

* S A (red triangles): This group shows a positive linear correlation. The solid red regression line starts at approximately 15 on the y-axis and rises to over 30 at the 850 mark on the x-axis. Data points are scattered but generally higher than other groups.

* S I (green squares): This group shows a slight negative trend. The dashed green regression line starts near 12 on the y-axis and descends slightly toward 8 at the far right of the x-axis.

* M D (blue circles): This group also shows a negative trend. The dotted blue regression line starts near 11 on the y-axis and descends toward 5 at the far right of the x-axis.

Most data points for all groups are clustered between negative 100 and 200 percent testosterone change, with a few outliers for each group extending toward the 900 mark.

Navigate back to Image 1:.

